# The inducible secreting TLR5 agonist, CBLB502, enhances the anti-tumor activity of CAR133-NK92 cells in colorectal cancer

**DOI:** 10.20892/j.issn.2095-3941.2023.0033

**Published:** 2023-09-19

**Authors:** Xiaohui Wang, Wei Qiu, Haoyu Liu, Min He, Wei He, Zhan Li, Zhiqiang Wu, Xiang Xu, Ping Chen

**Affiliations:** 1 College of Biotechnology, Southwest University, Chongqing 400715, China; 2 Department of Stem Cell & Regenerative Medicine, State Key Laboratory of Trauma, Burn and Combined Injury, Daping Hospital, Army Medical University, Chongqing 400042, China; 3 Department of Dermatology, The First Affiliated Hospital of Wenzhou Medical University, Wenzhou 325035, China; 4 Department of Biotherapeutics, The First Medical Center, Chinese PLA General Hospital, Beijing 100038, China

**Keywords:** TLR5 agonist, CBLB502, CAR133, endogenous immune response, colorectal cancer

## Abstract

**Objective:**

CAR-T/NK cells have had limited success in the treatment of solid tumors, such as colorectal cancer (CRC), in part because of the heterogeneous nature of tumor-associated antigens that lead to antigen-negative relapse after the initial response. This barrier might be overcome by enhancing the recruitment and durability of endogenous immune cells.

**Methods:**

Immunohistochemistry and flow cytometry were used to assess the expression of CD133 antigen in tissue microarrays and cell lines, respectively. Retroviral vector transduction was used to generate CBLB502-secreting CAR133-NK92 cells (CAR133-i502-NK92). The tumor killing capacity of CAR133-NK92 cells *in vitro* and *in vivo* were quantified *via* LDH release, the RTCA assay, and the degranulation test, as well as measuring tumor bioluminescence signal intensity in mice xenografts.

**Results:**

We engineered CAR133-i502-NK92 cells and demonstrated that those cells displayed enhanced proliferation (9.0 × 10^4^ cells *vs*. 7.0 × 10^4^ cells) and specific anti-tumor activities *in vitro* and in a xenogeneic mouse model, and were well-tolerated. Notably, CBLB502 secreted by CAR133-i502-NK92 cells effectively activated endogenous immune cells. Furthermore, in hCD133+/hCD133− mixed cancer xenograft models, CAR133-i502-NK92 cells suppressed cancer growth better than the counterparts (*n* = 5, *P* = 0.0297). Greater T-cell infiltration was associated with greater anti-tumor potency (*P* < 0.0001).

**Conclusions:**

Armed with a CBLB502 TLR5 agonist, CAR133-NK92 cells were shown to be capable of specifically eliminating CD133-positive colon cancer cells in a CAR133-dependent manner and indirectly eradicating CD133-negative colon cancer cells in a CBLB502-specific endogenous immune response manner. This study describes a novel technique for optimizing CAR-T/NK cells for the treatment of antigenically-diverse solid tumors.

## Introduction

Colorectal cancer (CRC) is the third leading cause of cancer-related deaths globally^1,2^. Despite significant advances in the treatment of CRC, the morbidity rate continues to increase, while the 5-year survival rate remains low^3,4^, highlighting the need to investigate modified or novel strategies for the treatment of CRC patients. Adoptive immunotherapy, which involves genetically-modified lymphocytes to produce chimeric antigen receptor-modified T cells (CAR-T), has emerged as one of the most promising techniques in cancer treatment^5^. Several organizations have published preclinical and clinical studies in which CRC was treated using CAR-T cells^6–8^. Unfortunately, the therapeutic effects of CAR-T cells in CRC have been unsatisfactory. This lack of efficacy is assumed to be due, at least in part, to antigen-negative relapse, which is caused by tumor association antigen (TAA) heterogeneity and antigen loss or downregulation^9^. Developing techniques to treat antigen-negative recurrence would thus provide a significant treatment edge. It was recently reported that increased density and durability of endogenous immune cells, in addition to transplanted CAR-T cells, overcomes this barrier and boosts CAR-T cell therapeutic efficacy in solid tumors^10,11^.

Toll-like receptor (TLR) agonists have been shown to be potent co-stimulatory signals as well as significant activators of the immune response, suggesting a viable way to improve the efficacy of cancer immunotherapies^12–14^; however, because of the acute inflammatory response, which poses significant safety concerns, the FDA has currently approved only two TLR agonists (the TLR4 agonist, monophosphorylated lipid A, and the TLR7 agonist, imiquimod)^15^. Currently, there is strong interest in developing the TLR5 agonist, flagellin, which is the only known natural ligand of TLR5, as an excellent candidate adjuvant for immunotherapy for several reasons. First, compared to other TLR agonists, flagellin is well-tolerated and generally safe because acute inflammation and cytokine storms do not occur during the TLR5 physiologic response^16,17^. Second, TLR5 signaling activation protects normal tissues from radiation and ischemia-reperfusion damage and enhances regeneration. Third, flagellin binds to the extracellular domain of TLR5, which is expressed on the surfaces of a variety of immune cells, including dendritic cells (DCs), macrophages, lymphocytes, and natural killer (NK) cells. Binding of flagellin to the extracellular domain of TLR5 triggers an MyD88-dependent signaling cascade and activates the nuclear factor NF-κB and mitogen-activated protein kinase pathways that regulate the innate and adaptive immune responses, which is thought to be important for adaptive immune response development^18,19^. Fourth, flagellin is the only known TLR agonist that can be engineered to be expressed and secreted by T cells; the majority of known TLR agonists consist of lipids or nucleic acids^20,21^. Fifth, flagellin has been shown to increase survival in tumor-bearing mice^22,23^.

A number of TLR5 agonists have been shown to stimulate the immune response in recent years. CBLB502, also known as entolimod, is widely used for both animal and clinical investigations. The drug toxicity of CBLB502 has been determined and reported in a clinical study in which cancer patients and healthy individuals were administered CBLB502 in parallel. CBLB502 was shown to be safe and well-tolerated (http://clinicaltrials.gov/ct2/show/NCT02654938). CBLB502 function was demonstrated based on the findings of a preclinical investigation in which CBLB502 was shown to mediate the immune response against cancer cells^24^. These findings prompted us to ascertain whether the TLR5 agonist, CBLB502, could boost CAR-T cell therapeutic efficacy against CRC. Several studies have shown that to ensure a sufficient quantity of efficacious TLR agonist reaches the tumor site, TLR agonists are typically delivered systemically at large doses, resulting in an excessive release of pro-inflammatory factors. We used TRUCKs (fourth-generation CAR-T cells) with modified protein release after CAR signaling in the current investigation^25^ to determine if CAR-NK cells selectively depositing CBLB502 at the tumor site could serve as a co-stimulatory tumor environment to restore anticancer activity.

In addition to mobilizing endogenous immune cells, eliminating cancer stem cells (CSCs) is another important strategy for increasing CAR-T cell therapy efficacy in CRC since colon cancer stem cells (CCSCs) are thought to be the primary source of cancer progression and recurrence^26,27^. As a specific molecular biomarker for CCSCs, CD133 is regarded as a reasonable target for immunotherapy^28,29^. Several clinical trials have revealed that targeting CD133 CAR-based immunotherapy is safe and well-tolerated^30,31^. This finding provided the impetus to investigate the therapeutic efficacy of anti-CD133 CAR-NK cells in colon cancer.

An alternative strategy to overcome the problem of antigen-negative tumor cells relapsing to CAR-T cells involves the use of NK cells instead of T cells as CAR vectors^32^. CAR-NK cells, unlike CAR-T cells, can be activated by CAR-independent mechanisms, such as the activated NKG2D receptor^33^, and can possibly eliminate tumor cells *via* CD16-mediated antibody-dependent cellular cytotoxicity (ADCC)^34^. Thus, the hypothesis for this study was that CAR-NK cells can successfully eradicate CAR-targeted antigen-positive and -negative tumor cells *via* CAR- and NK-cell receptor-dependent processes, respectively^35^. Moreover, NK cells are not limited to autologous cells. Several investigations have fully investigated NK92 cells (the NK cell line), as the CAR driver in preclinical and clinical studies^36–40^; however, the therapeutic potential in CRC has not been investigated. These findings pave the way for co-expression of the TLR5 agonist, CBLB502, in CAR133-NK92 cells to build an appealing treatment strategy in CRC.

In this study we engineered CAR133-NK92 cells with an inducible secreted CBLB502 (i502) expression cassette under the control of nuclear factor of activated T cell (NFAT)-IL2 minimal promoter (CAR133-i502-NK92). We demonstrated that CAR133-i502-NK92 cells exhibit enhanced proliferation and activation, which significantly suppressed tumor growth and prolonged survival in hCD133+ and hCD133+/hCD133− mixed colon cancer xenograft mouse models. We also showed that this impact is heavily reliant on T cell mobilization. Furthermore, we discovered that CBLB502 release from CAR133-i502-NK92 cells had a greater impact on immune cell activation.

## Materials and methods

### CD133 expression analysis in tissue microarrays and colon cancer cell lines

To measure the expression of CD133 in normal and colon cancer tissues, the HOrg-N090-PT-02 microarray (Outdo Biotech, Shanghai, China) containing 24 normal human tissue samples and the HColA-180Su11 microarray (Outdo Biotech) containing 76 colon cancer tissue samples were used to determine the expression of CD133 by immunohistochemistry (IHC), as previously described^41^. Briefly, after deparaffinization and rehydration, sections were exposed to 3% H_2_O_2_ in methanol to eliminate endogenous peroxidase activity. Bovine serum albumin [BSA (1%)] was used to block the sections for 30 min at room temperature (RT). The sections were then incubated with anti-human monoclonal antibodies against CD133 (ab19898; Abcam, Cambridge, UK) overnight at 4°C. The sections were then thrice-washed (5 min each) with 1X PBS and incubated with peroxidase-conjugated secondary antibodies (ChemMateTM DAKO EnVision™ Detection Kit, peroxidase/DAB, rabbit/mouse; DAKO, Agilent Technologies, Santa Clara, CA, USA) for 45 min at RT. Immunoreactivity of CD133 was detected in the cytoplasm and on cytoplasmic membranes. The level of CD133 expression was evaluated semi-quantitatively using the H score, which was calculated by multiplying the intensity of the signal by the relative strength. Low and high CD133 expression was scored using the median value as the cut-off threshold. Staining results were independently evaluated by two trained investigators. As a negative control, slides were processed as described above without the addition of primary antibodies. Slides that were used as positive controls for detection of the primary antigens were provided by Outdo Biotech.

To evaluate the surface expression of CD133 in colon cancer cell lines (SW620, SW480, and HCT116), the cells were washed with 1X PBS containing 4% BSA and incubated with PE-conjugated anti-CD133 antibody (ab253271; Abcam); IgG isotype controls were included. The stained cells were analyzed using a NovoCyte flow cytometer (ACEA Biosciences, Hangzhou, China) and CellQuest Pro software (BD Biosciences, San Jose, CA) according to the manufacturers’ protocols.

### Cell lines and culture conditions

Human CRC (SW480 and SW620), mouse CRC (CT26), and human monocytic cell lines (THP-1) were obtained from the American Type Culture Collection (Manassas, VA, USA). The human CRC cell line, HCT116, was kindly provided by Jian Yu (University of Pittsburgh, Pittsburgh, PA, USA). The human NK cell line, NK92, was provided by Jianhua Yu (Ohio State University, Columbus, OH, USA). Human monocytic THP-1 cells were cultured in Roswell Park Memorial Institute medium (RPMI-1640; Invitrogen, Carlsbad, CA, USA) containing 10% FBS and 2 mmol/L L-glutamine (Gibco, Carlsbad, CA, USA) and differentiated into M0 macrophages by incubation for 24 h with 150 nM phorbol 12-myristate 13-acetate (PMA; Sigma, St. Louis, MO, USA). NK92 cell lines were cultured in alpha minimum essential medium (α-MEM; Gibco) supplemented with 12.5% heat-inactivated fetal bovine serum (FBS; Gibco), 12.5% horse serum (Gibco), and 150 IU/mL recombinant human interleukin-2 (IL-2; GenScript, Nanjing, Jiangsu, China). CRC cell lines (SW480 and SW620) were transduced using a retroviral vector encoding the firefly luciferase (FFluc) gene, as previously described^42^. All cell lines were cultured at 37°C in a 5% CO_2_ incubator.

Human T cells were isolated from healthy donors with informed consent and expanded *in vitro*, as previously described^43^. Briefly, peripheral blood mononuclear cells (PBMCs) were collected from patients by leukapheresis. Primary T cells derived from PBMCs were activated using Dynabeads Human T-Activator CD3/CD28 magnetic beads (Invitrogen, Carlsbad, CA, USA) at a bead:cell ratio of 1:1 and cultured in X-VIVO 15 medium (Lonza, Basel, KB, Switzerland) supplemented with 300 U/mL of recombinant human IL-2 (PeproTech, Rocky Hill, NJ, USA).

### Generation of CAR133-i502-NK92 cells

All CAR designs were 4-1BB (CD137)-based second generation. CAR133 containing anti-CD133 scFv was derived from GenBank accession no. HW350341.1, human CD137, and CD3ζ signaling domains, as previously described^31^. To design an anti-CD133 CAR that produces the TLR5-based agonist, CBLB502, in an inducible manner and to selectively deposit CBLB502 into the tumor tissue while avoiding systemic immune cell activation, we took advantage of fourth generation CAR-T cells engineered with protein or cytokine release, following CAR signaling (also called TRUCK)^25^. Specifically, the conventional CAR133 construct was further engineered to include an inducible TLR5 agonist CBLB502 (i502) expression cassette under control of the nuclear factor of activated T cell (NFAT)-IL2 minimal promoter (i502-CAR133). The use of such an expression construct means that CBLB502 is released by the engineered NK92 cells following CAR engagement of cognate antigens. To facilitate the analysis, CBLB502 was expressed as a translational fusion polypeptide with an oligo-His-tag, which has been shown to have little, if any, effect on protein binding affinity or anti-cancer capability^44^. The CBLB502 sequence, which is composed of the conserved N- and C-terminal domains required for TLR5 activation, is from the flagellin of *Salmonella enterica* serovar Dublin (GenBank accession no. M84973.1)^45^. The two CARs with CD133 specificity were inserted into a pWPXL lentiviral vector using *Bam*HI and *Nde*I enzymes. The entire sequence was then confirmed by direct sequencing. CAR133-i502-NK92 cells were generated using standard techniques, as previously described^37,46,47^. Transduction was performed in 24-well plates using 2 × 10^6^ NK cells/500 μL of lentiviral supernatant containing polybrene (8 μg/mL) and recombinant human interleukin-2 [rhIL-2 (250 U/mL)], and infected in the incubator for 12 h. The cells were further centrifuged and incubated overnight, after which the old supernatant was removed and fresh medium was added.

The surface expression of CARs on the transduced NK92 cells was analyzed by flow cytometry, which was performed as previously described^38,39^. Briefly, transduced NK cells were washed with PBS containing 4% BSA and incubated with biotin-labeled goat anti-mouse F(ab′)2 polyclonal antibody or with normal polyclonal goat IgG antibody as an isotype control.

### Proliferation, cytotoxicity, and exhaustion analysis

To measure the proliferation of effector cells, CAR133-NK92 and CAR133-i502-NK92 cells were co-cultured with the mitomycin C-treated CD133-positive colon cancer cell line, SW620, and the CD133-negative colon cancer cell line, SW480. Effector cells were harvested, and the cell number was counted on days 3, 5, and 7 using a Cell Counting Chamber slide (Invitrogen), according to the manufacturer’s instructions.

To assess the cytotoxicity of effector cells, two CD133-positive colon cancer cell lines (SW620 and HCT116) and one CD133-negative colon cancer cell line (SW480) were co-cultured with effector cells at an effector-to-target ratio (E:T) of 10:1. After incubation of the cells for 24 h, culture supernatant was obtained from each sample, and LDH levels were measured using an LDH cytotoxicity kit (Promega, Madison, WI, USA) according to the manufacturer’s instructions. The cytotoxicity of effector cells was also evaluated using the RTCA assay with an xCELLigence RTCA TP system (ACEA Biosciences, Hangzhou, China), as previously described^48^. Briefly, CAR133-NK92 and CAR133-i502-NK92 cells were added at a 10:1 ratio to the wells of an E-Plate 16 that had been seeded 24 h earlier with various target cells, and target cell proliferation was monitored by measuring the conductivity of the cell surface area in contact with the gold electrodes covering the plate surface. In addition, degranulation of the effector cells was analyzed by detecting the cell surface expression of CD107a using the BD FastImmune CD107a Kit (BD Biosciences). Briefly, anti-human CD107a antibody was added to the medium in which CAR133-NK92 and CAR133-i502-NK92 cells were co-cultured with target cells at an E:T of 10:1. One hour later, 1 μL of GolgiStop (BD Biosciences) was added, and the cultures were incubated for an additional 4 h. Effector cells were collected, and CD107a expression was analyzed using a BD FACS Canto II flow cytometer and CellQuest Pro software. All experiments were performed in triplicate.

To analyze genes encoding activity receptors, inhibitory receptors, and exhaustion genes, CAR133-NK92 and CAR133-i502-NK92 cells were co-cultured with SW620 cells at an E:T ratio of 10:1 for 3 days. The effector cells were then collected and washed, and total RNA was extracted using the RNAsimple Total RNA Kit (Tiangen, Beijing, China) according to the manufacturer’s instructions. The cDNA was synthesized using a PrimeScript^®^ RT Reagent kit (Takara, Otsu, Shiga, Japan), and expression of the exhaustion markers (PD-1, TIM-3, and LAG-3), activity receptors (NKp46, NKp44, and NKG2D), and the inhibitory receptor (NKG2A) was measured in triplicate using SYBR Green qPCR Master Mix (Thermo Fisher, Waltham, MA, USA) and the Bio-Rad CFX Manager system. β-actin was used as an endogenous control. The primers used in this study are listed in **Supplementary Table S1**.

### Conditioned media (CM) derived from CAR133-i502-NK92 cells effectively activated NK cells, T cells, and macrophages

CAR133-NK92 and CAR133-i502-NK92 cells were co-cultured with SW620 cells that had been treated for 2 h with 10 μg/mL of mitomycin C at an E:T ratio of 10:1 without IL-2 in RPMI-1640 medium containing 10% FBS and 2 mmol/L L-glutamine (Gibco). Three and 5 days later, conditioned medium from the 2 groups was collected and stored at −20°C for use in further stimulation of immune cells. THP-1 cells were differentiated from M0 macrophages by exposure to 150 nM PMA for 24 h. T cells, NK92 cells, and macrophages were seeded into 6-well plates (1 × 10^5^ cells/well) and cultured in X-VIVO 15 medium, α-MEM, and RPMI-1640 medium, respectively. After 24 h, the medium was replaced with CM, and the cells were cultured for an additional 24 h and propagated without further stimulation in the absence of exogenous cytokines or feeder cells. To evaluate the role of CBLB502 in induced activation and proliferation protein expression, CMs were pre-incubated with an anti-TLR5 monoclonal antibody at 1 μg/mL (Invitrogen, Carlsbad, CA, USA) for 1 h based on the manufacturer’s instructions. Then, T cells, NK92 cells, and macrophages were collected and washed twice with PBS containing 4% BSA. Immunostaining was performed using anti-human Ki67-APC, CD69-APC, CD86-PE, and CD206-FITC, and isotype controls according to a standard surface immunostaining flow cytometry protocol. The stained cells were counted using a NovoCyte flow cytometer (ACEA Biosciences) according to the manufacturer’s protocol. The data were analyzed using CellQuest Pro software. Expansion of NK92 cells was calculated using the Cell Counting Kit-8 (CCK-8; Beyotime, Shanghai, China) according to the manufacturer’s instructions.

### Microarrays for cytokine release

Human cytokine array C5 (Ray Biotech, Atlanta, GA, USA) was used according to the manufacturer’s instructions to qualitatively measure the cytokines and chemokines released by CAR133-NK92 and CAR133-i502-NK92 cells co-cultured with SW620 cells that had been treated for 24 h with 10 μg/L of mitomycin C for 3 and 5 days. Briefly, the array membranes were blocked for 30 min, incubated overnight with 100 μL of samples at 4°C, then incubated with biotin-conjugated antibodies at RT for 2 h. The membranes were then washed and incubated with HRP-conjugated secondary antibody for 2 h. Finally, the membranes were incubated with chemiluminescent substrate and exposed to X-ray film for 15 min. Chemiluminescence measurements of the arrays were quantified using ImageJ; the fold change over the control is shown in heatmaps. The ratios between the two groups were calculated based on three experiments. Individual quantification of granulocyte-macrophage colony-stimulating factor (GM-CSF), IL-7, and IL-6 was performed using specific ELISA kits.

### *In vivo* anti-tumor studies

#### Xenogeneic mouse model

A xenograft colon cancer model was established in 6- to 8-week-old BALB/c nude mice, purchased from the Model Research Center of Nanjing University (Nanjing, China). Mice were subcutaneously inoculated in the left flank on day 0 with 1 × 10^6^ SW620 cells labeled with firefly luciferase (FFluc) or 5 × 10^5^ SW620 cells in combination with 5 × 10^5^ SW480 tumor cells labeled with FFluc. Tumor-bearing host mice were not subjected to pre-conditioning or lymphodepletion before effector cell treatment. On day 14 after transplantation, the tumor volume reached approximately 100-200 mm^3^, and mice were randomly divided into 3 groups for injection with NK92, CAR133-NK92, or CAR133-i502-NK92 cells (*n* = 5 each) based on the tumor bioluminescence signal intensity (BLI) to ensure a similar tumor burden among the experimental groups. Mice were injected intravenously (*i.v.*) with effector cells at a dose of 5 × 10^6^ cells/mouse on days 0, 3, and 6. Mice treated with NK92 cells were used as the control group. During this period, each mouse was injected with 1,000 U of IL-2 every other day. For the SW620 and SW480 mixed cell population mouse model, mice were randomly divided into four groups for injection with CAR133-NK92 cells, CAR133-i502-NK92 cells, pre-T cells + CAR133-NK92 cells, or pre-T cells + CAR133-i502-NK92 cells (*n* = 5 each). In the two pre-T-cell groups, before injection with effector cells, the mice were pre-injected with 1 × 10^5^ human T cells. Mouse body weight was measured twice per week. BLI of tumor-bearing mice was performed by injecting the mice intraperitoneally with D-luciferin potassium salt (150 mg/kg; Beyotime) dissolved in PBS and quantifying luminescence using the FUSION FX imaging system (Vilber Lourmat, Paris, France). Luminescence images were collected and analyzed using Fusion software (Vilber Lourmat). The mice were monitored for survival and euthanized when exhibiting signs of distress. All mice were euthanized when the tumor volume in the control mice crossed the cut-off volume of 1,500 mm^3^.

#### Analysis of effector cell persistence in mouse peripheral blood

Mouse peripheral blood samples were collected by retro-orbital exsanguination. Cell pellets were isolated by centrifugation of the collected blood at 5,000 rpm for 5 min. Red blood cells were lysed in 0.45 mL 1× BD FACS Lysing Solution (BD Biosciences) and 50 μL of each sample was then incubated with CD56-PerCP antibody while being protected from exposure to light for 15 min at RT. The proportion of effector cells was analyzed using a BD FACS Canto II flow cytometer and CellQuest Pro software.

#### H&E staining and IHC analysis

Tumor tissues and organs, including the heart, liver, kidneys, lungs, and stomach, were harvested from the tumor-bearing mice, fixed in formalin, embedded in paraffin, and prepared as 2-mm thick sections. The sections were mounted on slides and histopathologically-stained with H&E. Tumor tissue sections were stained for infiltration by human T cells using a mouse monoclonal antibody against human CD3ε (Thermo Scientific, Waltham, MA, USA) and for tumor angiogenesis using a mouse monoclonal antibody against human CD31 (Abcam). Following overnight incubation with the primary antibody at 4°C, the secondary antibody was added, and the results were visualized using a ChemMate Envision Detection Kit (DakoCytomation, Carpinteria, CA, USA).

### Study approval

This study was performed in accordance with the Declaration of Helsinki and was approved by the Ethics Committee for Human Subject Research of Southwest University (Chongqing, China) and Shanghai Outdo Biotech Company (No. YB M-05-02). The peripheral blood samples were collected with informed written consent and was approved by the Ethics Committee of the Daping Hospital of Army Medical University (REC No. 2020-151). All animal experiments were performed in accordance with the Animal Care and Use Committee guidelines of the NIH (Bethesda, MD, USA) and were conducted under protocols approved by the Institutional Animal Care and Use Committee of Daping Hospital and the Research Institute of Surgery.

### Statistical analysis

Data are presented as the mean ± SD. A two-tailed *t*-test was utilized to compare significant differences between means. A *P* value < 0.05 was considered statistically significant. For microarray data, nSolver analysis software was used to identify proteins and cytokines that displayed statistically significant differential expression using a *t*-test that assumes unequal variance. For *in vivo* bioluminescence intensity, the Kruskal-Wallis test was utilized to compare the median of the CAR133-i502-NK92 group to that of the CAR133-NK92- and NK92-treated groups. For survival data, Kaplan–Meier curves were plotted, and the differences between groups were analyzed using a log-rank test. Prism 7.0 (GraphPad) software was used to perform the statistical calculations.

## Results

### CD133 is upregulated expression in CRC tissues

**Figure 1:**
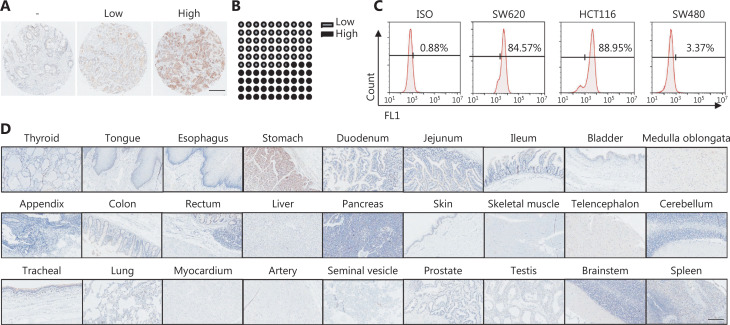
CD133 has upregulated expression in colorectal cancer tissues. (A) The HColA-180Su11 microarray (Outdo Biotech, Shanghai, China) containing 76 primary colon cancer samples was stained with an anti-CD133 antibody to determine the expression of CD133. Left: paracarcinoma tissue with negative CD133 expression; middle: primary colon cancer samples with lower CD133 expression; right: primary colon cancer samples with higher CD133 expression. Representative images (magnification ×4) are shown for CD133 immunostaining. The scale bar represents 500 μm. (B) The percentage of different levels of CD133 expression is indicated. (C) CD133 expression on colon cancer cell lines was analyzed by flow cytometry. (D) The HOrgN090PT02 microarray (Outdo Biotech) containing 25 human normal tissue samples was immunostained with an anti-CD133 antibody to determine the expression of CD133; human normal tissues are negative for CD133. Representative staining image fields (magnification ×20) are shown. Scale bars represent 100 μm.

We first assessed CD133 expression in primary colon cancer tissues by performing immunohistochemical staining of tissue microarrays containing 76 samples. As expected, CD133 was positively expressed in all tissues and was highly expressed in 43.4% of the samples (**Figure 1A, 1B**). Next, we determined CD133 expression in normal human tissues by using a tissue microarray containing 25 types of tissue samples. Positive staining was only noted in the stomach tissues (**Figure 1C**). Additionally, CD133 expression in the colon cancer cell lines (HCT116, SW480, and SW620) was examined by flow cytometry. CD133 was highly expressed in HCT116 and SW620 cells (88.95% and 84.57%, respectively), but not in SW480 cells (**Figure 1D**). Therefore, in subsequent experiments we considered SW480 cells as the control group, while SW620 and HCT116 cells were the experimental groups.

### Generation of CBLB502-secreting CAR133-NK92 cells

**Figure 2:**
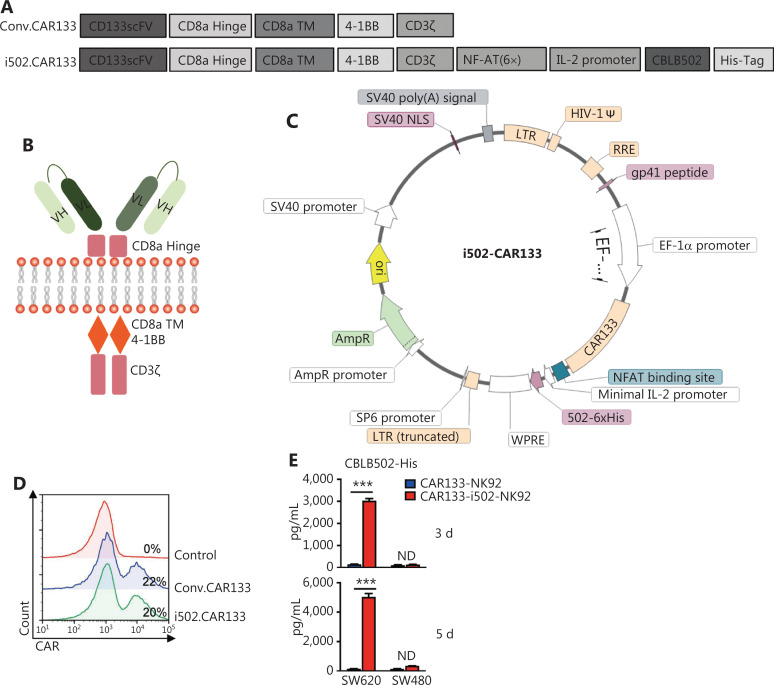
Construction and characterization of CAR133-i502-NK92 cells. (A and B) Schematic diagram of the second-generation conventional (Conv) and i502-CAR gene constructs are shown. (C) The i502-CAR gene was cloned into the BamHI and NdeI sites of an EF1-alpha promoter-based lentiviral vector (pWPXL). (D) Levels of CAR expression in transduced NK92 cells were analyzed by flow cytometry after the cells were stained with an anti-Fab antibody or IgG1 isotype control. NK92 cells not transduced with the pWPXL lentiviral vector were used as a negative control. (E) CAR133-NK92 and CAR133-i502s-NK92 cells were co-cultured with SW620 cells for 3 and 5 days. As negative controls, CAR133-NK92 cells were co-cultured with SW480 cells. CBLB502 in the culture supernatants was examined for production using ELISA (mean ± SD, *n* = 3, ***P < 0.001), ND, not detected.

A second generation CD133-specific CAR (conv. CAR133) containing anti-CD133 scFv, the CD8α-hinge, the transmembrane domain of CD8α (CD8α-TM), the co-stimulatory molecule, 4-1BB, and the human CD3ζ domain was successfully constructed, as previously described^30,31^ (**Figure 2A, 2B**). The CARs with CD133 specificity were inserted into a pWPXL lentiviral vector using *Bam*HI and *Nde*I enzymes (**Figure 2C**). The entire sequence was then confirmed by direct sequencing.

NK92 cells were expanded according to the standard procedure and stably transduced with the two constructs (conv. CAR133 and i502-CAR133) to generate CAR133-NK92 and CAR133-i502-NK92 cells, respectively. CAR expression in CAR133-i502-NK92 cells was almost equivalent to or slightly lower than CAR expression in CAR133-NK92 cells (**Figure 2D**). Secretion of CBLB502 by CAR133-i502-NK92 cells was assessed using ELISA, which showed that CAR133-NK92 cells without any additional modification did not secrete CBLB502 either spontaneously or after CAR stimulation (**Figure 2E**). In contrast, CBLB502 was secreted by CAR133-NK92 cells engineered with CBLB502 when the cells were co-cultured with hCD133+ SW620 cells, but not in the presence of hCD133-SW480 cells, which indicated that CAR133-i502-NK92 cells initiated CBLB502 release when exposed to CD133-positive tumor cells.

### CBLB502 enhanced CAR133-NK92 cell proliferation, activation, and anti-tumor activity *in vitro*

**Figure 3:**
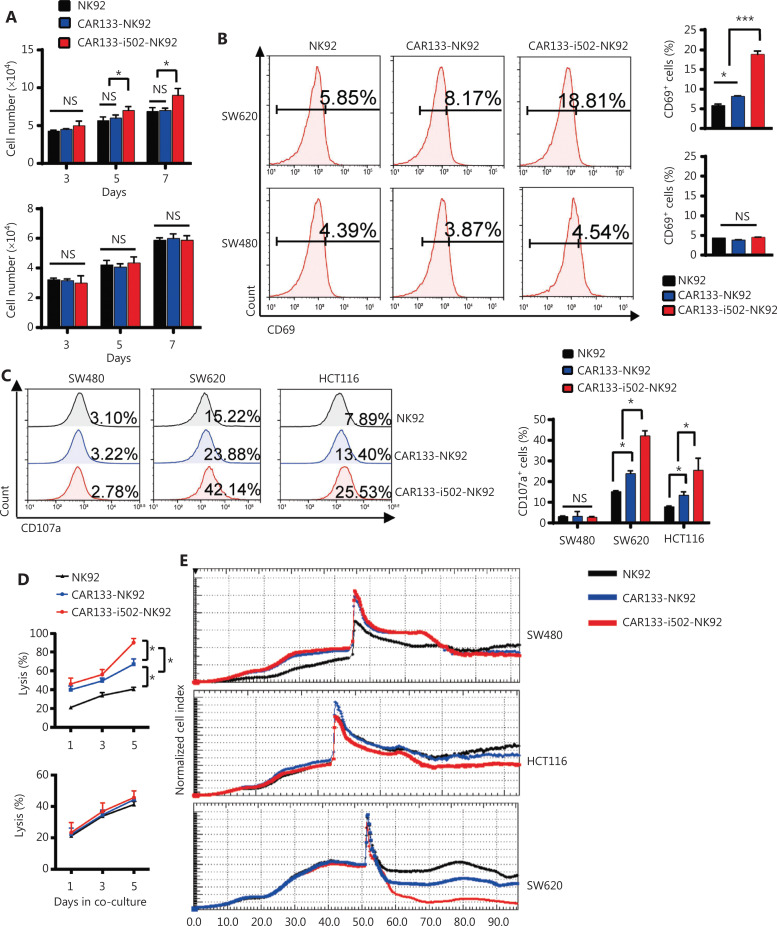

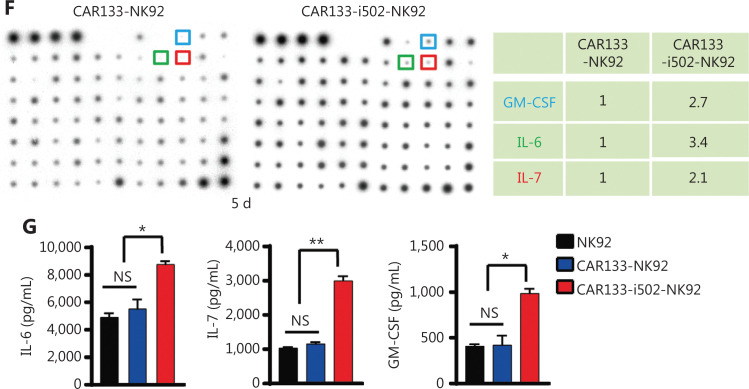
CAR133-NK92 cells with inducible secretion of CBLB502 enable superior proliferation, activation, and antitumor activity. (A) The absolute number of CAR133-NK92 and CAR133-i502-NK92 cells was analyzed when the cells were stimulated with CD133+ and CD133− antigens for different lengths of time (days). Data represent the mean ± SD of quadruplicate wells, *P < 0.05; NS, not significant. (B) The activation marker, CD69, was quantified by flow cytometry when CAR133-NK92 or CAR133-i502-NK92 cells were cultured with SW620 and SW480 cells for 24 h. The mean values from triplicate cultures are shown. ***P < 0.001. (C) Effector cells (NK92, CAR133-NK92, and CAR133-i502-NK92 cells) were co-cultured with colorectal cancer cells at an E:T ratio of 10:1 for 24 h. Effector cells were then harvested and CD107a expression was analyzed by flow cytometry. The mean values from triplicate cultures are shown. *P < 0.05; **P < 0.01; NS, not significant. (D) Cell killing by CAR133-NK92 and CAR133-i502-NK92 cells was investigated by the LDH cytotoxicity assay after co-culture with target cells at the indicated E:T ratios. All data are expressed as the means ± SEMs of triplicate samples. *P < 0.05; NS, not significant. (E) The cytotoxicity of CAR133-NK92 and CAR133-i502-NK92 cells against colon cancer cells was calculated by RTCA. (F) CAR133-NK92 and CAR133-i502-NK92 cells were co-cultured with SW620 cells for 5 days, and the cytokines and chemokines in the culture supernatant were determined by the human cytokine array C5 (Ray Biotech, Atlanta, GA, USA). *P < 0.05. (G) The concentrations of GM-CSF and IL-7 in the supernatant were further determined by specific ELISA kits. Data represent the mean+SEM. *P < 0.05; **P < 0.01.

It has been reported that T cells engineered to secrete flagellin exhibit increased cell proliferation^49^; therefore, we first evaluated the proliferation of CAR133-i502-NK92 cells and observed that the proliferation of NK92, CAR133-NK92, and CAR133-i502-NK92 cells was comparable when the cells were co-cultured with hCD133 + SW620 cells for 3 days. CAR133-i502-NK92 cells exhibited enhanced proliferaton compared to CAR133-NK92 cells following co-cultivation with hCD133+ SW620 cells for 7 days (*P* = 0.0176; **Figure 3A**). When the CAR133-i502-NK92 cells were co-cultured with hCD133-SW480 cells, little difference was detected among NK92, CAR133-NK92, and CAR133-i502-NK92 cells with respect to cellular expansion on different days (*P* > 0.05).

Next, we showed that CAR133-i502-NK92 cells displayed enhanced expression of the activation marker, CD69, compared to NK92 and CAR133-NK92 cells following co-cultivation with hCD133+ SW620 cells (*P* < 0.001). When the three effector cells were co-cultured with hCD133-SW480 cells, this phenomenon disappeared (*P* > 0.05; **Figure 3B**). Likewise, compared to CAR133-NK92 cells, the expression of active receptors, such as NKp46, NKp44, and NKG2D, was significantly enhanced, while inhibitory receptors, such as NKG2A, were significantly decreased on CAR133-i502-NK92 cells following co-cultivation with hCD133+ SW620 cells; no difference in markers associated with NK cell exhaustion (PD-1, TIM-3, and LAG-3) was observed (**Supplementary Figure S1A**). Upon CD133− antigen stimulation, neither activation/inhibition receptors nor exhaustion proteins were differentially expressed in CAR133-NK92 or CAR133-i502-NK92 cells (**Supplementary Figure S1B**). To assess anti-tumor activity, we showed that CAR133-NK92 and CAR133-i502-NK92 cells exhibited enhanced CD107a expression compared to non-transduced NK92 cells when co-cultured with hCD133+ SW620 and hCD133+ HCT116 cells, while the three effector cells had similar levels of CD107a expression when co-cultured with hCD133-SW480 cells. Moreover, compared to CAR133-NK92 cells, the level of CD107a expression on CAR133-i502-NK92 cells was significantly higher (*P* < 0.001; **Figure 3C**). The cytolytic function of effector cells was further assessed by LDH and RTCA, and both CAR133-NK92 and CAR133-i502-NK92 cells displayed enhanced tumor lytic potential against hCD133+ SW620 and HCT116 cells compared to NK92 cells, but showed equal lytic potential against hCD133-SW480 cells. Compared to CAR133-NK92 cells, CAR133-i502-NK92 cells mediated significant cell lysis (**Figure 3D, 3E**). Overall, these data suggest that co-expression of CAR133 and CBLB502 enhanced NK92 cell proliferation, activation, and anti-tumor activity.

Furthermore, we characterized the cytokine profile in the supernatants of CAR133-NK92 cells and CAR133-i502-NK92 cells when stimulated with SW620 cells. The production of cytokines was generally comparable between CAR133-NK92 and CAR133-i502-NK92 cells for 3 days (**Supplementary Figure S2A**); however, compared to CAR133-NK92 cells, CAR133-i502-NK92 cells exhibited increased production of pro-inflammatory cytokines, such as granulocyte-macrophage colony-stimulating factor (GM-CSF), IL-6, and IL-7, 5 days after tumor stimulation (**Figure 3F**). GM-CSF, IL-6, and IL-7 concentrations in the culture supernatants were further measured using ELISA (*P* < 0.001; **Figure 3G, Supplementary Figure S2B**). Because NK-92 cells do not usually produce IL-6, we performed Western blotting to determine the expression of IL-6 in SW620 cells that were co-cultured with CAR133-NK92 and CAR133-i502-NK92 cells, respectively. A higher IL-6 level in SW620 cells co-cultured with CAR133-i502-NK92 cells was shown compared to SW620 cells co-cultured with CAR133-NK92 cells (**Supplementary Figure S2C**).

### CAR133-i502-NK92 cells delayed tumor growth in mice with subcutaneous hCD133-expressing colon tumors

**Figure 4:**
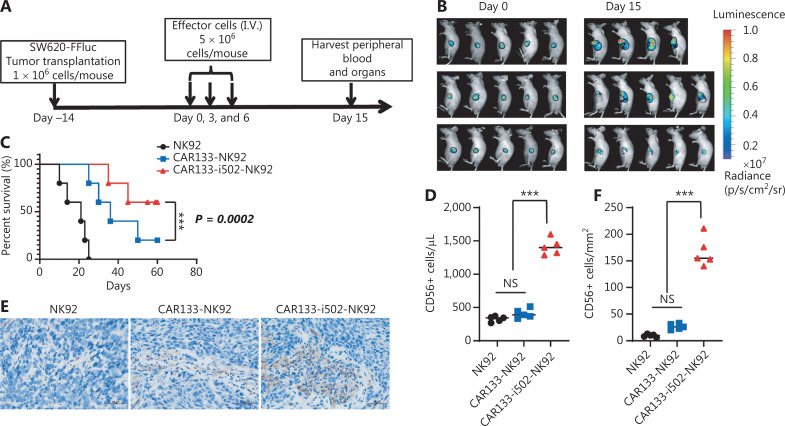
CBLB502 promotes the antitumor activity of CAR133-NK92 cells in mice with subcutaneous CD133-expressing colon tumors. (A) Schedule of the challenge protocol. Mice were inoculated with CD133+ SW620 cells (1 × 10^6^ cells/mouse) followed 14 days later by CAR133-i502-NK92, CAR133 NK92, or NK92 cells (5 × 10^6^ cells/mouse, *n* = 5/group). (B) Colon cancer growth was monitored by *in vivo* bioluminescence imaging. (C) Kaplan–Meier survival curves showing the tumor-free survival of tumor-bearing mice treated with CAR133-i502-NK92, CAR133-NK92, or NK92 cells. Significance was determined by the log-rank test. ***P < 0.001. (D) Blood collected on day 15 was analyzed by flow cytometry for the presence of effector cells, CAR133-i502-NK92 cells showed enhanced proliferation *in vivo*. ***P < 0.001. (E) The number of CD56+ cells, representing NK92 cells, was increased in tumor samples of mice treated with CAR133-i502-NK92 cells compared to CAR133-NK92 and NK92 cells on day 15 after effector cell treatment. Each scale bar represents 50 μm. (F) Absolute number of CD56+ cells in **Figure 4E**. Data represent the mean ± SD of quadruplicate wells. ***P < 0.001.

Encouraged by our observation that CAR133-i502-NK92 cells exhibited enhanced anti-tumor activity *in vitro*, we investigated the anti-tumor activity in a mouse xenograft tumor model, which was constructed through engraftment of mice with hCD133+ SW620 cells labeled with FFluc. The experimental design is shown in **Figure 4A**. Compared to control cells, CAR133-i502-NK92 and CAR133-NK92 cells significantly controlled tumor progression by day 15. Compared to CAR133-NK92 cells, CAR133-i502-NK92 cells exerted more potent anti-tumor effects (**Figure 4B, Supplementary Figure S3**). CAR133-i502-NK92 cell therapy also significantly prolonged mouse survival at the time of sacrifice, compared with the other two groups (*P* < 0.001; **Figure 4C**). Moreover, blood collected on day 15 was analyzed by flow cytometry for the presence of effector cells. The number of CAR133-i502-NK92 cells in the blood of mice injected with those cells was elevated 3-fold to 1,680 cells/μL compared to the number of CAR133-NK92 cells in the blood of mice injected with CAR133-NK92 cells (*P* < 0.001; **Figure 4D**). To assess tumor infiltration of effector cells, tumor samples were collected 15 days after adoptive transfer of effector cells and the presence of NK92 cells was assessed using immune histology. The number of CD56+ cells, representing NK92 cells, was increased in tumor samples that were treated with CAR133-i502-NK92 cells compared to the other two groups (*P* < 0.001; **Figure 4E, 4F**).

**Figure 5:**
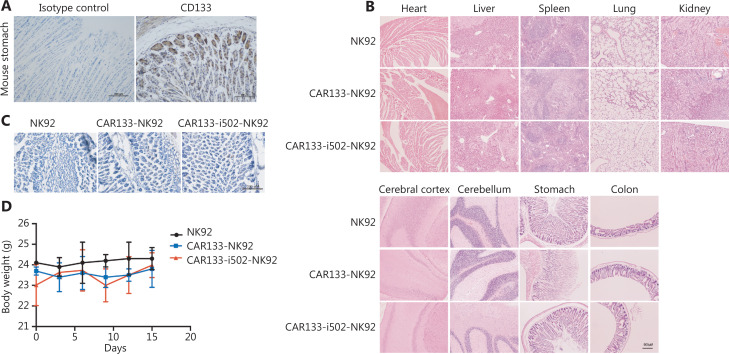
On-target off-tumor toxicities in the mouse model treated with CAR133-i502-NK92 cells. (A) The expression of mouse CD133 protein was tested in mouse stomach tissue using IHC assays. Representative staining image fields (magnification ×200) are shown. Scale bars represent 100 μm. (B) Histopathologic analysis of mouse organ tissues by H&E staining. Magnification ×20. Each scale bar represents 500 μm. (C) Detection of CAR-NK92 cells infiltrated into stomach tissues on day 15 after effector cell treatment by IHC staining of CD56 showed that there was minimal immune cell infiltration into the stomach (magnification ×100), The scale bar represents 200 μm. (D) Body weight of the individual mice from each treatment group. Data are presented as the mean ± SD of three independent experiments. Error bars represent the standard deviation.

Because CD133 was positively stained in human stomach tissues, we examined CD133 expression in mouse stomach tissues; a high level of CD133 expression was observed (**Figure 5A**). We further determined whether murine CD133 activated CAR133-i502-NK92 cells. Enhanced expression of CD69 was observed on CAR133-i502-NK92 cells following co-cultivation with mCD133+ CT26 cells for 24 h (**Supplementary Figure S4**). Therefore, it was necessary to determine whether CAR133-i502-NK92 cells were toxic to mouse stomach tissues. Following a CAR133-i502-NK92 cellular infusion, a detailed pathologic examination of the mice was also performed when euthanized on day 15. Various organs and tissues, including the heart, liver, spleen, lungs, and kidneys, were extracted and stained with H&E; no pathologic abnormalities or mononuclear cells were observed in any of the tissues (**Figure 5B**). In addition, we performed IHC staining of CD56 in the stomach tissues after treatment and showed that there were only a few CAR-NK92 cells that had infiltrated the stomach tissues (**Figure 5C**). Taken together, the results of the H&E and IHC staining indicated that CAR133-NK cells were well-tolerated *in vivo* and had very low off-target toxicity in the stomach. We also analyzed CAR-NK92 cell distribution in the stomach and tumor, and showed that the number of CAR-NK92 cells infiltrating into the tumor tissues was significantly higher than the stomach tissues (**Supplementary Figure S5**). Further analysis indicated that mice treated with CAR133-i502-NK92 cells exhibited transient weight loss on day 8, but this was not significant. The weight returned to baseline levels and remained stable thereafter (**Figure 5D**). All these data support the notion that CAR133-i502-NK92 cells enhanced anti-tumor activity, but did not induce on-target, off-tumor toxicity in tumor-bearing nude mice.

### NK cells, T cells, and macrophages were effectively activated by CM from CAR133-i502-NK92 cells

**Figure 6:**
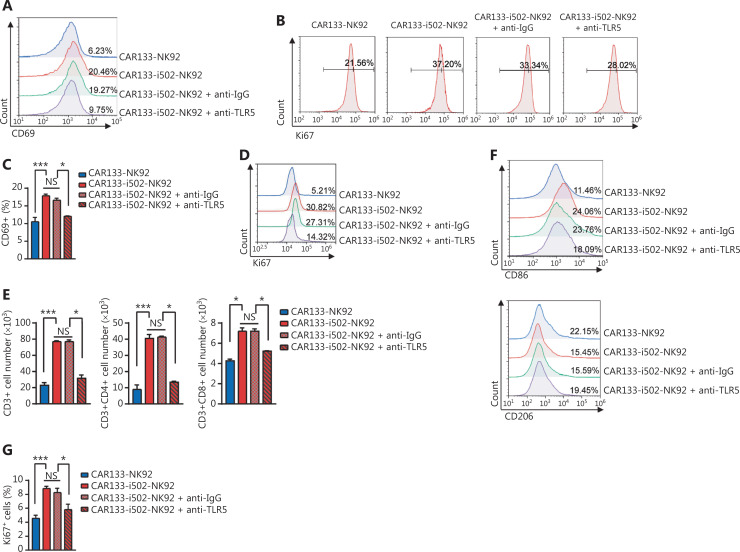
CBLB502 secretion from CAR133-i502-NK92 cells effectively activate NK cells, T cells, and macrophages. (A and B) CM derived from CAR133-i502-NK92 cells significantly enhanced expression of the activation marker, CD69 (A) and the proliferation marker, Ki67 (B) on NK92 cells compared to CM derived from CAR133-NK92. ***P < 0.001. (C and D) CD69 (C) and Ki67 (D) expression on T cells was assessed by flow cytometry when T cells were stimulated with CM from CAR133-NK92 or CAR133-i502-NK92 cells or with an anti-TLR5 monoclonal antibody. Experiments were repeated three times and representative histograms are shown. Data represent the mean ± SEM. *P < 0.05, ***P < 0.001. (E) Absolute number of CD3+, CD3+CD4+, and CD3+CD8+ T subpopulations was significantly increased when T cells were stimulated with CM from CAR133-i502-NK92 cells. Experiments were repeated three times and representative histograms are shown. Data represent the mean ± SD of quadruplicate wells. *P < 0.05, ***P < 0.001. (F) CD86+ and CD206+ macrophages were analyzed when macrophages were stimulated with CM from CAR133-NK92 or CAR133-i502-NK92 cells or using an anti-TLR5 monoclonal antibody by flow cytometry. *P < 0.05, ***P < 0.001. (G) Ki67 expression on macrophages was assessed by flow cytometry. Experiments were repeated three times, and representative histograms are shown. Data represent the mean ± SEM. *P < 0.05, ***P < 0.001.

CBLB502 activates the immune response by recognizing receptors expressed on lymphocytes, NK cells, macrophages, and other cells. Therefore, we characterized the endogenous immune cell response to CBLB502 secreted from CAR133-i502-NK92 cells. We showed that beyond CBLB502, the production of cytokines was generally comparable in CM derived from CAR133-NK92 and CAR133-i502-NK92 cells stimulated with CD133 antigen for 3 days. Therefore, we collected the above CM, which were used to stimulate NK92 cells, primary human T cells, and macrophages. As expected, administration of CM derived from CAR133-i502-NK92 cells significantly enhanced expression of the activation marker, CD69 (*P* < 0.001; **Figure 6A, Supplementary Figure S6A**), and the proliferation marker, Ki67 (*P* < 0.001; **Figure 6B, Supplementary Figure S6B**), on NK92 cells compared to CM derived from CAR133-NK92 cells. To confirm the role of CBLB502 signaling, we added an anti-TLR5 neutralizing antibody to CM derived from CAR133-i502-NK92 cells to block CBLB502 signaling and showed that the enhanced proliferation and activation activity of CBLB502 in NK92 cells was inversely changed, highlighting the role of CBLB502 in promoting NK92 cell activation and proliferation (**Supplementary Figure S6C**).

Consistent with the above data, compared to CM derived from CAR133-NK92 cells, CM derived from CAR133-i502-NK92 cells also significantly enhanced CD69 (*P* < 0.001; **Figure 6C, Supplementary Figure S7A**) and Ki67 expression (*P* < 0.001; **Figure 6D, Supplementary Figure S7B**) on primary human T cells, while the number of CD3+, CD3+CD4+, and CD3+CD8+ subpopulations was significantly increased (**Figure 6E**). Moreover, the CD4+:CD8+ T-cell ratio did not differ between the two groups (**Supplementary Figure S7C**). In addition, the enhanced proliferation and activation was inversely changed when an anti-TLR5 neutralizing antibody was added to CM from CAR133-i502-NK92 cells. These results indicate that CBLB502 release from CAR133-i502-NK92 enhanced the proliferation and activation capacity of T cells.

Subsequently, we tested the role of CBLB502 in regulating the expression of M1- and M2-type macrophage-related proteins. As shown in **Figure 6F** and **Supplementary Figure S8A, S8B**, the level of CD86 expression (*P* < 0.001; M1-type macrophages) was increased, while the level of CD206 expression (*P* < 0.001; M2-type macrophages) was decreased in cells treated with CM derived from CAR133-i502-NK92 cells compared to cells treated with CM derived from CAR133-NK92 cells, demonstrating that CBLB502 hasthe potential to stimulate macrophages to polarize towards a pro-inflammatory phenotype. Macrophages also upregulated the expression of Ki67 when treated with CM derived from CAR133-i502-NK92 cells compared to CM derived from CAR133-NK92 cells (*P* < 0.001; **Figure 6G, Supplementary Figure S8C**). Similarly, the enhanced proliferation and activation activities were inversely changed when an anti-TLR5 neutralizing antibody was added to the CM from CAR133-i502-NK92 cells.

### CBLB502 significantly enhanced the anti-tumor activity of CAR133-NK92 cells in hCD133+/hCD133- mixed colon cancer xenograft mouse models

**Figure 7:**
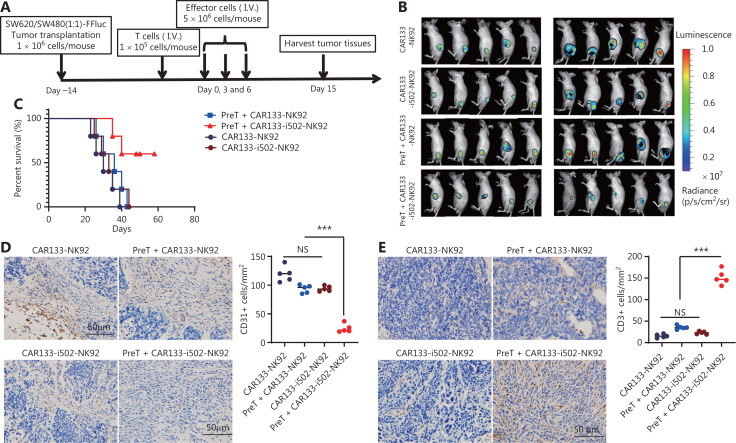
Adoptively transferred CAR133-i502-NK92 cells successfully control tumor development in mice with CD133+ and CD133− mixed colon tumor models. (A) Schedule of the *in vivo* experimental design using CD133+ and CD133- mixed colon tumor model. (B) BLI illustrating colon cancer growth. (C) Kaplan–Meier survival curves generated from the survival of mice treated with CAR133-i502-NK92 and CAR133-NK92 cells. ***P < 0.001. (D) Expression of CD31 protein was tested in the mouse tumor tissue using IHC assays. Representative photomicrographs are shown (magnification ×400). The scale bar is 50 μm. ***P < 0.001. (E) Representative immunostaining images of CD3+ T-cell infiltration into cancer tissues. Representative staining of tumor sections in each experimental group is shown (magnification ×400). The scale bar represents 50 μm. ***P < 0.001.

**Figure 8:**
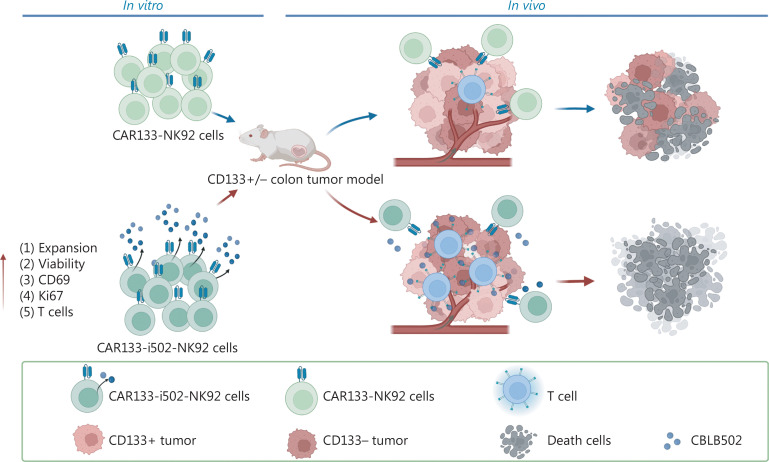
The schematic diagram of the anti-tumor activity of CAR133-NK92 cells in colorectal cancer. We engineered CAR133-NK92 cells with inducible secretion of CBLB502 and demonstrated that CAR133-i502-NK92 cells not only specifically eliminate CD133-positive colon cancer cells in a CAR133-dependent manner, but also indirectly eradicate CD133-negative colon cancer cells in a CBLB502-specific endogenous immune response manner. The figure was created with Biorender.com.

To mimic the clinically relevant pathophysiology of a heterogeneous tumor with a substantial number of cancer cells lacking the targeted antigen, we inoculated nude mice with a mixed population of equal amounts of hCD133+ and hCD133− colon cancer cells, we determined the expression of CD133 in tumor tissues on days 7 and 14 after transplantation of a SW620 and SW480 cell mixture by IHC staining. As shown in **Supplementary Figure S9A**, although the CD133+:CD133− ratio *in vivo* was not exactly 1:1 during the modeling stage, possibily because the growth discrepancy was also observed *in vitro*, these cells remained in a heterogeneous state that mimicked the pathophysiology of solid tumors. These mice were then treated with CAR133-NK92 cells. The experimental design is shown in **Figure 7A**. Neither CAR133-i502-NK92 nor CAR133-NK92 cells were effective in controlling cancer progression, and there were no significant differences between the two groups. The presence of T cells did not further improve the beneficial effect of CAR133-NK92 cells; however, T-cell supplementation not only enhanced the anti-tumor effect (**Figure 7B, Supplementary Figure S9B**), but also prolonged the survival of mice with CAR133-i502-NK92 cells (*P* < 0.001; **Figure 7C**). We next detected CD133+ cancer cells remaining in tumor tissues by IHC staining of CD133 in the tumor samples collected 15 days after treatment and showed that the majority of residual tumor cells were CD133-negative, but there were also residual CD133-positive tumor cells (**Supplementary Figure S9C**). In addition, CD31, a marker of tumor angiogenesis, was also shown to have low expression in mouse tumor tissues from the group that had CAR133-i502-NK92 cells preinjected with T cells compared to tissues from the other three groups (**Figure 7D**). CAR133-i502-NK92 cells supplemented with T cells maintaining anti-tumor effects in such mixed tumors might be attributed to mobilization by the CBLB502 signal to T cells. To test this hypothesis, we perfomred H&E experiments to analyze T-cell infiltration into tumor tissues and showed that tumor tissues derived from mice treated with CAR133-i502-NK92 cells preinjected with T cells induced massive CD3+ T-cell infiltration, which was substantially more than that observed in the other three groups (*P* < 0.001; **Figure 7E**). To exclude the possibility that TLR5 expression in colon cancer cells might affect the antitumor activity of CRC treatment, we performed IHC staining of TLR5 in human normal colon and primary colon cancer tissues and showed that TLR5 had a lower level of expression in primary colon tumor and normal colon tissues (**Supplementary Figure S9D**). By performing flow cytometry, TLR5 was shown to have lower expression in SW620 and SW480 cell lines (**Supplementary Figure S9E**). Therefore, the antitumor effect of CAR133-i502-NK92 cells in this model should not be affected by the low TLR5 expression on colon cancer cells. In addition, we measured the weights of mice that underwent therapeutic regimens and showed that there were no significant differences in body weights among the four groups (**Supplementary Figure S9F**). Our findings suggest that the use of an appropriate combination of CAR133 with the immune regulatory factor TLR5 agonist, CBLB502, enhanced the anti-tumor potential of NK92 cells in coordination with the mobilization of tumor-reactive endogenous immune cells (**Figure 8**).

## Discussion

CAR-T/NK92 cells were shown to be a promising cancer immunotherapy strategy; however, the efficacy of CAR-T/NK92 cells remains limited by antigenic heterogeneity. We utilized CBLB502 as an immune adjuvant to engineer CAR133-NK92 cells and demonstrated that CAR133-NK92 cells secreting immune modulatory CBLB502 not only exhibited enhanced amplification, activation, and cytotoxicity to tumor cells *in vitro*, but also exhibited better anti-tumor responses *in vivo*. It has been reported that the increased anti-tumor activity of flagellin-secreting T cells may be associated with elevated levels of cytokines and chemokines^49^. We also showed significantly increased IL-6, IL-7, and GM-CSF levels in the supernatants of CAR133-i502-NK92 cells co-cultured with tumor cells. Pro-inflammatory cytokine (GM-CSF and IL-6) secretions were demonstrated to be TLR5-dependent and respond to CBLB502 in a dose-dependent manner. Krivokrysenko et al.^50^ also suggested that G-CSF and IL-6 may be candidate biomarkers for the efficacy of CBLB502 *in vivo*. This idea was further supported by Eremina et al.^51^, who reported that Mobilan, consisting of human Toll-like receptor 5 and CBLB502, induced elevated G-CSF and IL-6 levels. We are of the opinion that CBLB502 binds to TLR5 and induces the production of inflammatory cytokines by activating NF-κB signaling, thus enhancing the activity of CAR133-i502-NK92 cells. In addition, we also showed elevated IL-7 production in the supernatant, which has a key role in promoting cell proliferation and inhibiting apoptosis of NK cells^52,53^. This finding may explain why CAR133-i502-NK92 cells exhibited enhanced proliferation ability compared to CAR133-NK92 cells without CBLB502.

We then demonstrated the important role of CBLB502 secretion from CAR133-i502-NK92 cells in the activation of endogenous immune cells. Flagellin has been reported to activate the immune response by recognized receptors expressed on lymphocytes, NK cells, macrophages, and other cells^54–57^. Activation of TLR5 signaling has also been shown to increase the expression of CD83, CD80, and CD86, thus transforming tolerant dendritic cells into activated antigen-presenting cells^58,59^. We found that NK cells, T cells, and macrophages are effectively activated by CBLB502 secreted from CAR133-i502-NK92 cells. In a recent study, Kaczanowska and Davila^49^ reported that intratumoral delivery of flagellin-producing T cells offer a therapeutic benefit by altering several aspects, including augmenting T-cell effector function and expansion, as well as reducing the number of T-cell exhaustion markers. Our study extended this finding and further showed that CBLB502 secreted by CAR133-i502-NK92 cells not only promoted CAR133-i502-NK92 cell proliferation but also elicited the endogenous immune cell response; however, the underlying mechanism has not been established. We hypothesized that TLR5 is the main signaling molecule that mediates CBLB502 to activate the immune response. Indeed, when TLR5 signaling was blocked, the ability to activate endogenous immune cells among CAR133-i502-NK92 cells was also inhibited.

The focus of our study was to analyze the anti-tumor effect of CAR133-i502-NK92 cells in tumors with antigen heterogeneity or loss. To mimic the clinical heterogeneity of solid tumors, we used a hCD133+/hCD133− mixed colon cancer xenograft mouse model, which has been proven to be viable in solid tumor studies. We observed inhibition of tumor growth and improved survival in xenogeneic mouse models treated with CAR133-i502-NK92 cells followed by pre-injection of T cells compared to CAR133-i502-NK92 cells without pre-injection of T cells, which might be associated with the mobilization of T cells that eradicate hCD133− colon tumor cells. Indeed, an increased number of CD3+ immune cells was shown to infiltrate into the tumor tissues of mice treated with CAR133-i502-NK92 cells followed by pre-injection of T cells. CBLB502 mobilization of anti-tumor immune cells has been emphasized by several groups. Among them, Rhee and colleagues^23^ demonstrated that flagellin regulates neutrophil infiltration, thereby controlling the immune responses to tumors. Burdelya^60^ showed that CBLB502 induces numerous immunomodulatory factors, recruits various types of immune cells, and inhibits the growth and liver metastasis of multiple tumors. Yang and colleagues^17^ demonstrated that CBLB502-driven recruitment of NK cells to the liver is critical for the anti-tumor efficacy in tumor models. Our study extended these findings and broadened the use of CBLB502 in CAR-NK92 cell immunotherapy.

One challenge that cannot be ignored in CAR-T/NK-cell therapy is safety, where on-target off-tumor cytotoxicity represents an overt life-threatening danger. In 2010 a patient treated with anti-ERBB2-directed CAR-T cells developed multiorgan failure due to ERBB2 expression in normal lung cells^61^. The CD133 antigen that we used to target colon cancer was positively expressed in human and mouse normal stomach tissues and the CAR133 construct we used to specifically target human CD133 antigen was derived from a murine scFv; however, it is important to analyze CAR133-NK92 cell safety *in vivo*. In this study pathologic examination of mouse tissues (including the colon, brain, and stomach) revealed no signs of adverse events, suggesting that CBLB502 secreted by the CAR133-NK92 cells enhanced anti-tumor activity without conveying toxicity to normal tissues. There are two possible explanations for this unexpected phenomenon: the microenvironment of stomach tissues may be protective from the cytotoxicity of CAR133-NK92 cells; and the presence of CBLB502 might protect gastric tissue from toxic injury by CAR133-NK92 cells. In 2004 it was demonstrated that TLRs have a crucial role in the maintenance of intestinal epithelial homeostasis, while activation of TLRs by commensal microflora is critical for protecting against gut injury and associated mortality^62^. Jiang et al.^63^ reported similar results using claudin18.2 as a CAR-T cell target for stomach cancer, although claudin18.2 is expressed in stomach cancer and normal tissues. Jiang et al.^63^ found no off-target toxicity for anti-claud 18.2 CAR-T cells in normal stomach tissue. In addition, the safety of CD133-specific CAR-T cells has already been demonstrated in clinical studies, but no obvious off-target toxicities have been reported^30,31^.

Despite these promising findings, there are still some limitations to be considered. First, the immune deficiency mouse model we used lacks a functioning immune system to evaluate the efficacy of CAR133-i502-NK92 cells in a microenvironment similar to the clinical tumor microenvironment. We observed inhibition of tumor growth and improved survival in xenogeneic mouse models treated with CAR133-i502-NK92 cells followed by pre-injection of T cells, as well as increased infiltration of CD3+ immune cells into the tumor tissue, thus suggesting that T cells have an important role in this process. Therefore, further analysis of the effect of CAR133-i502-NK92 cells on bystander immune cells and modulation of the tumor microenvironment in the PDX model and immune reconstitution model is warranted. Second, because flagellin has been reported to prevent pre-existing tumor cells from developing into solid tumors at metastatic sites, it may be essential to determine whether CAR133-i502-NK92 cells prevent cancer metastasis and relapse. Third, although NK92 cell lines are considered a promising cellular tool for cancer immunotherapy, but because NK92 cells are tumors, NK92 cells must be irradiated prior to xenograft administration to avoid further cell proliferation *in vivo*.

## Conclusions

In summary, CAR133-NK92 cells armed with the TLR5 agonist, CBLB502, confer a new ability to mobilize immune cells and mediate an anti-tumor response to tumors that escape killing by CAR133-NK92 cells, which helps prevent tumor escape from CAR-T/NK cell immunotherapy. We also demonstrated that CAR-NK92 cells serve not only as direct anti-tumor effector cells, but also as cellular vectors to convey immune regulatory molecules to the tumor microenvironment. Adopting this approach, anti-tumor immune responses in solid tumors can be triggered, augmented, and sustained.

## Data Availability

The data generated in this study are available upon request from the corresponding author.
