# Supplementary material for: The inducible secreting TLR5 agonist, CBLB502, enhances the anti-tumor activity of CAR133-NK92 cells in colorectal cancer
Source: Cancer Biol Med. 2023 Sep 19;20(9):662–81. doi: 10.20892/j.issn.2095-3941.2023.0033 (PMC10546094; doi:10.20892/j.issn.2095-3941.2023.0033)
Supplement: Supplementary file 1 [file cbm-20-662-s001.pdf]

Supplementary materials

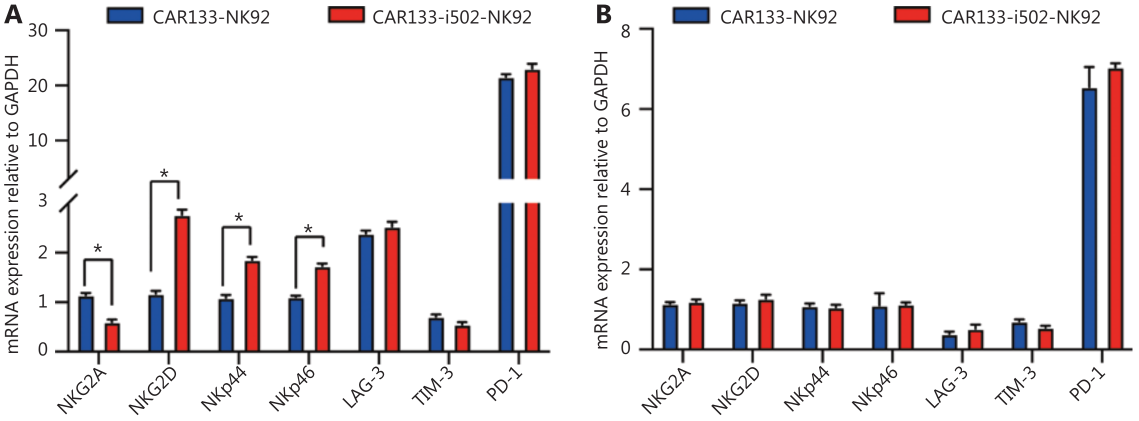

Figure S1 (A, B) CAR133-NK92 or CAR133-i502-NK92 cells were cultured with SW620 (A) and SW480 (B) cells for 3 days. mRNA expression of activity receptors, such as NKp46, NKp44, and NKG2D, inhibitory receptors, such as NKG2A, and NK-cell exhaustion markers, such as PD-1, TIM-3, and LAG-3, was assessed by RT-PCR. **P* < 0.05.

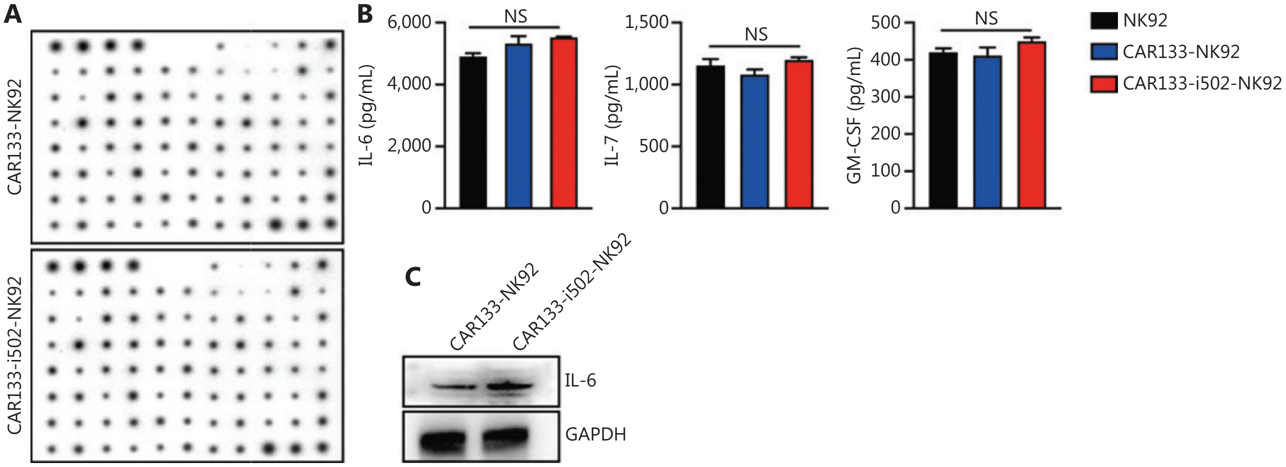

Figure S2 (A) CAR133-NK92 and CAR133-i502-NK92 cells were co-cultured with SW620 cells for 3 days, and the cytokines and chemokines in the culture supernatant were determined by the human cytokine array C5 (Ray Biotech, Atlanta, GA, USA). (B) The concentrations of IL-6, GM-CSF, and IL-7 in 3-day supernatants were determined using specific ELISA kits. Data represent the mean + SEM. (C) CAR133-NK92 and CAR133-i502-NK92 cells were co-cultured with SW620 cells for 5 days, and IL-6 expression in SW620 cells was determined by Western blotting, which showed a higher IL-6 level in SW620 cells co-cultured with CAR133-i502-NK92 cells compared to SW620 cells co-cultured with CAR133-NK92 cells.

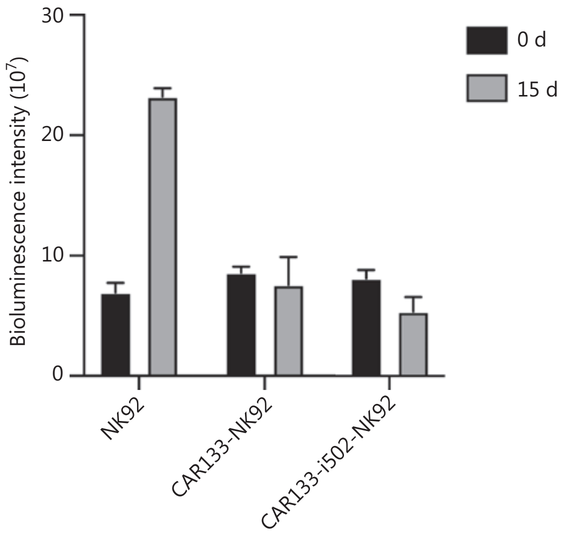

Figure S3 Bioluminescent flux plot quantifying tumor burden in response to different treatment groups over time.

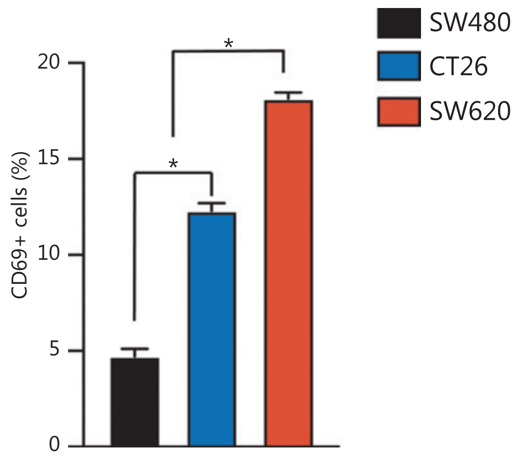

Figure S4 Expression of CD69 on CAR133-i502-NK92 cells was recorded by flow cytometry. Data represent the mean ± SEM. *$P < 0.05$.

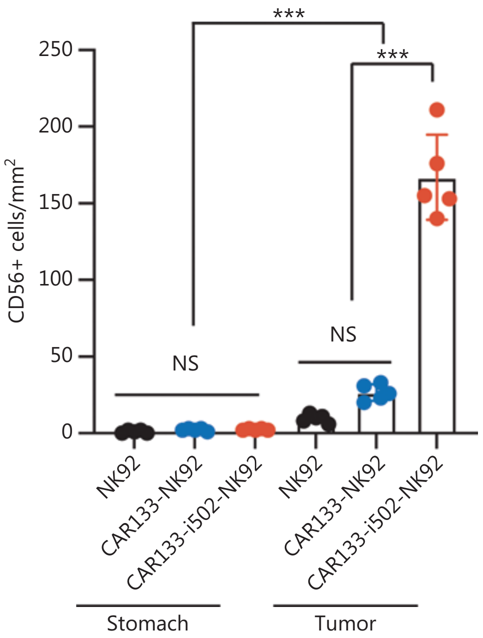

Figure S5 The absolute number of CD56+ cells in tumor and mouse stomach samples treated with CAR-NK92 cells was analyzed. The number of CAR-NK92 cells infiltrating tumor tissues was significantly higher than stomach tissues. ***$P < 0.001$.

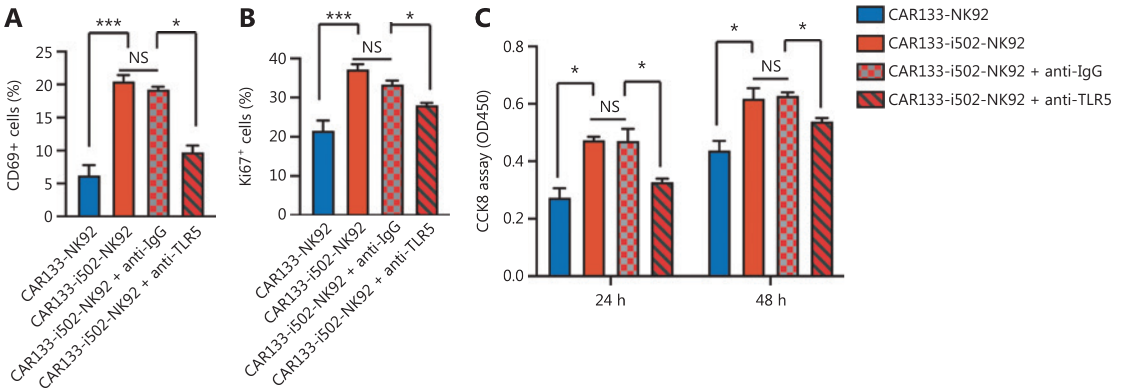

Figure S6 (A and B) Expression of CD69 and Ki67 on NK92 cells was recorded by flow cytometry. Data represent the mean ± SEM. ***$P < 0.001$; *$P < 0.05$. (C) Expansion of NK92 cells in the presence of CM from CAR133-NK92 or CAR133-i502-NK92 cells was analyzed using the CCK-8 assay. *$P < 0.05$.

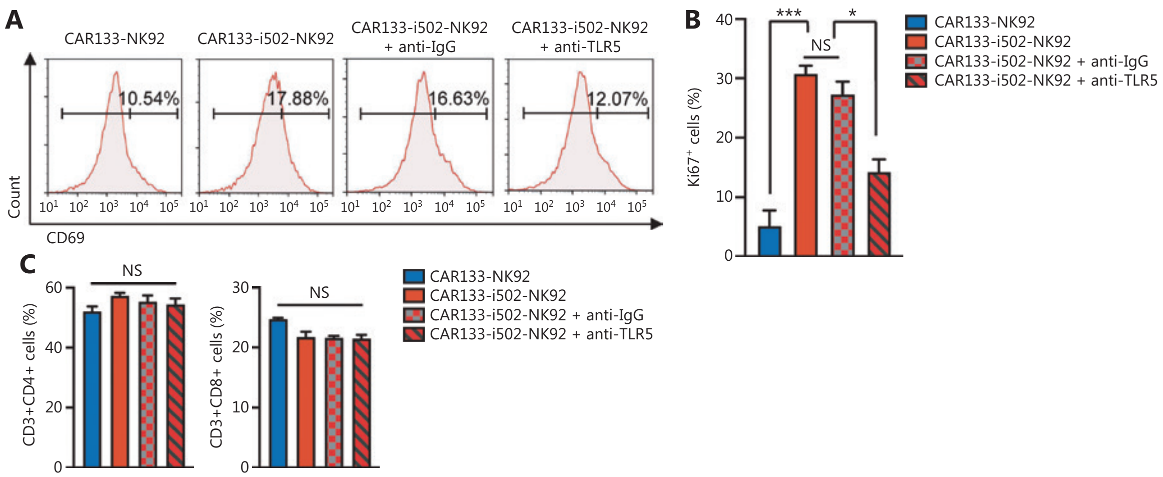

Figure S7 (A) Expression of CD69 on T cells was recorded by flow cytometry. (B) Ki67 expression on T cells was examined by flow cytometry after stimulation with CM from CAR133-NK92 or CAR133-i502-NK92 cells or with an anti-TLR5 monoclonal antibody for 24 h. Data represent the mean ± SEM. *$P < 0.05$, ***$P < 0.001$; NS, not significant. (C) The relative CD4+:CD8+ T-cell ratio of T cells after stimulation with CM from CAR133-NK92 or CAR133-i502-NK92 cells or stimulation with an anti-TLR5 monoclonal antibody for 24 h was assessed by flow cytometry; NS, not significant.

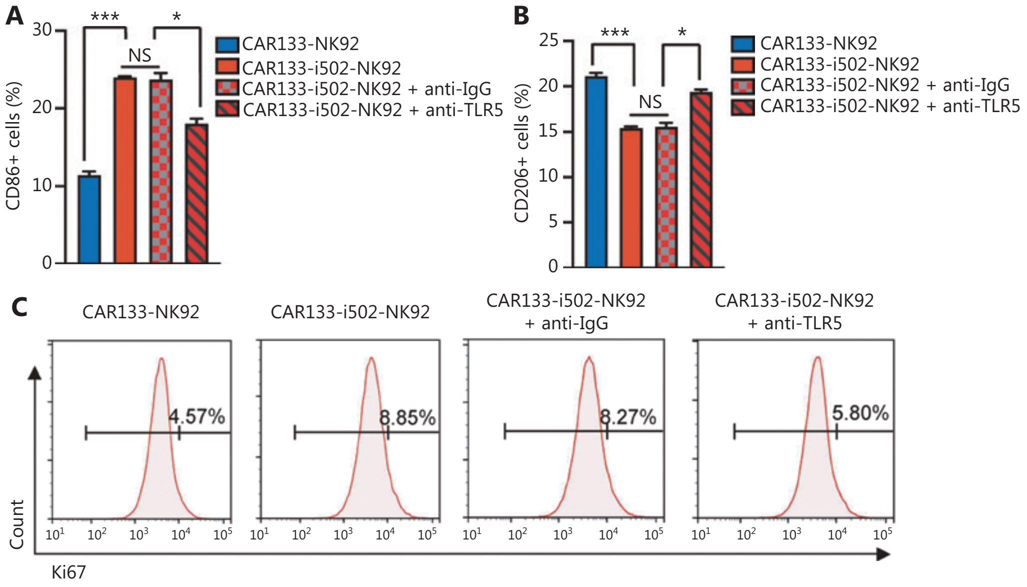

Figure S8 (A and B) CD86+ and CD206+ macrophages were analyzed by flow cytometry, Data represent the mean ± SEM. *P < 0.05, ***P < 0.001; NS, not significant. (C) Ki67 expression on macrophages was assessed by flow cytometry when cells were stimulated with CM from CAR133-NK92 or CAR133-i502-NK92 cells or with an anti-TLR5 monoclonal antibody for 24 h.

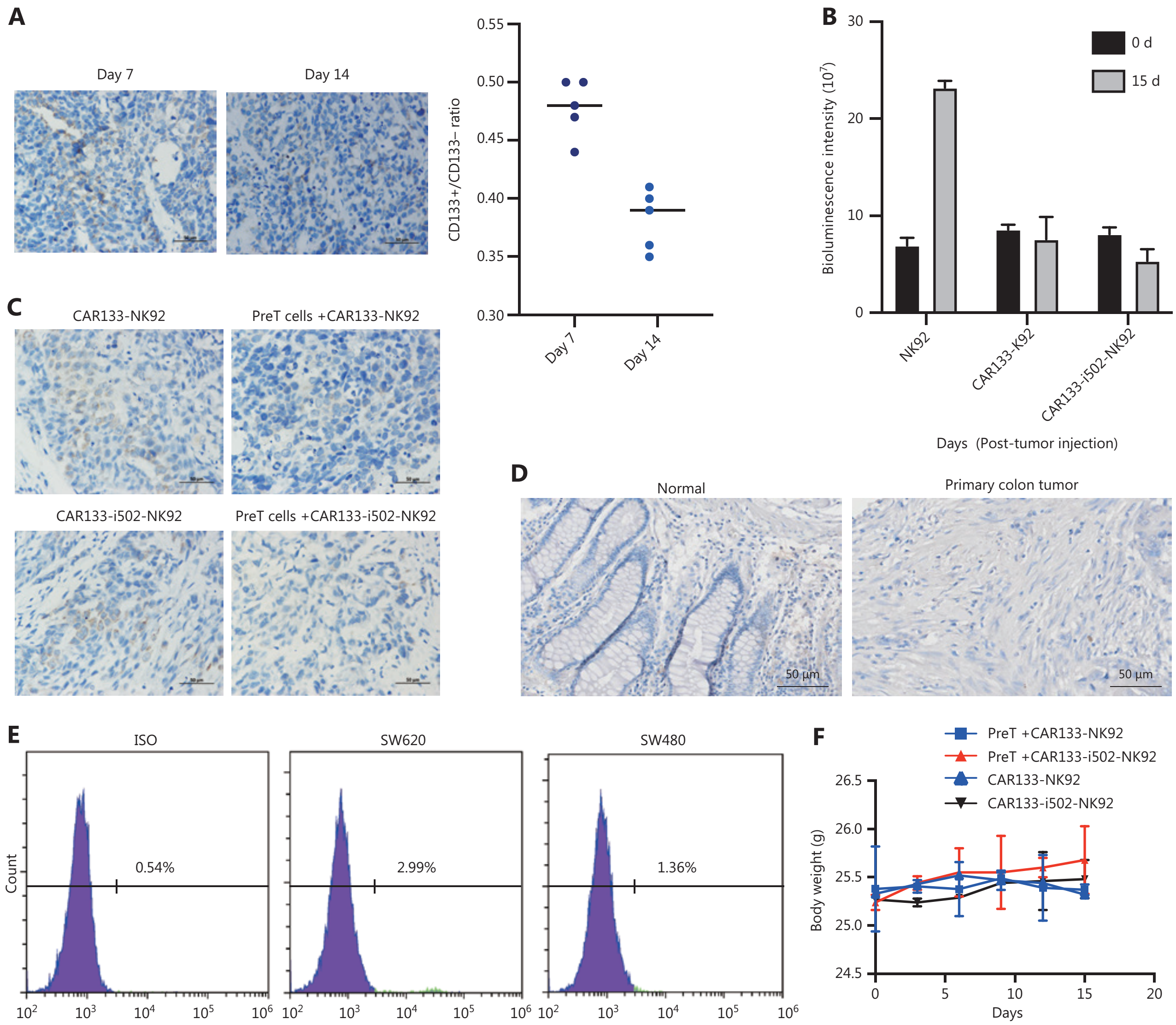

Figure S9 (A) IHC staining of CD133 in tumor tissues collected on days 7 and 14 after tumor cells transplantation showed that the CD133+:CD133− ratio *in vivo* was not 1:1 at the tumor modeling stage (magnification ×20). The scale bar represents 50 μm. (B) Bioluminescent flux plot quantifying tumor burden in response to different treatment groups over time. (C) Body weight of the individual mice from each treatment group. (D) IHC staining of TLR5 in human normal colon tissue and primary colon cancer tissue. TLR5 was less expressed in both primary colon tumor tissue and normal colon tissue (magnification ×20). The scale bar represents 50 μm. (E) Analysis of TLR5 expression in colon cancer cells by flow cytometry. TLR5 was also less expressed in colon cell lines of SW620 and SW480 cells. (F) Body weight of the individual mice from each treatment group. Data are presented as the mean ± SD of three independent experiments. Error bars represent the standard deviation.

Table S1 Primer sequences

Genes	Forward primer (5′-3′)	Reverse primer (5′-3′)
NKG2A	TTG CTG GCC TGT ACT TCG A	CCA AAC CAT TCA TTG TCA CCC
NKG2D	CTGGTGAAGTCATATCATTGGATGG	GCTCGAGGCATAGAGTGCACAG
NKp44	TACACCCACTGCTACTGCTGCTG	CACTTTGAAGTACCTGAGCCTTGGA
NKp46	TAACCACAGAGACGGGACTCCA	TGCTGAGCCAGTCTTCAACCA
PD-1	CCAGGATGGTTCTTAGACTCCC	TTTAGCACGAAGCTCTCCGAT
TIM-3	TCCAAGGATGCTTACCACCAG	GCCAATGTGGATATTTGTGTTAGATT
LAG-3	GCGGGGACTTCTCGCTATG	GGCTCTGAGAGATCCTGGGG
β-ACTIN	AGAGCTACGAGCTGCCTGAC	AGCACTGTGTTGGCGTACAG
